# Association of Body Mass Index with Echocardiographic Parameters and Incidence of Left Atrial Thrombus or Spontaneous Echo Contrast in Patients with Nonvalvular Atrial Fibrillation: A Cross-Sectional Study

**DOI:** 10.31083/RCM26014

**Published:** 2024-12-31

**Authors:** Yi Qiu, Shu Jiang

**Affiliations:** ^1^Department of Clinical Nutrition, The First People's Hospital of Changzhou, 213003 Changzhou, Jiangsu, China; ^2^Department of Echocardiography and Cardiology, The First People's Hospital of Changzhou, 213003 Changzhou, Jiangsu, China

**Keywords:** BMI, NVAF, echocardiographic, LAT/SEC

## Abstract

**Background:**

This article focuses on the effect of body mass index (BMI) on cardiac structure and function in cases with non-valvular atrial fibrillation (NVAF). Only a few articles have investigated the relationship between BMI and the incidence of left atrial thrombus (LAT) or spontaneous echo contrast (SEC) in cases with NVAF.

**Methods:**

This single-center retrospective study was conducted at The First People's Hospital of Changzhou. A total of 282 patients who were diagnosed with NVAF and planned to undergo radiofrequency ablation from 2019 to 2022 were enrolled in this study. None of the patients received standardized anticoagulant therapy. The patients were divided into a normal weight group, an overweight group, and an obesity group based on their BMI. The differences in echocardiographic parameters and LAT/SEC incidences among the three groups were compared, and regression analysis was applied to determine the correlation between BMI and the occurrence rates of LAT/SEC. The generalized additive model (GAM) was used to clarify the dose-response association between BMI and LAT/SEC.

**Results:**

Left atrial diameter (LAD), left ventricular end-diastolic diameter (LVEDD), interventricular septal thickness (IVST), left ventricular posterior wall thickness (LVPWT), left ventricular ejection fraction (LVEF), right atrial diameter (RAD), and the incidences of LAT/SEC were statistically different among the three groups. Univariate and multivariate logistic regression analyses indicated that BMI was related to the incidences of LAT/SEC. For each 1-unit increase in BMI, the odds of LAT/SEC increased by 12% (odds ratio (OR): 1.12, 95% CI: 1.02, 1.24). A threshold nonlinear relationship was found using the GAM between BMI and the risk of LAT/SEC.

**Conclusions:**

BMI significantly affects multiple echocardiographic parameters in patients with NVAF, and BMI is an independent risk factor for LAT/SEC in cases with NVAF.

## 1. Introduction

Atrial fibrillation (AF) is the most frequently encountered cause of cardiac arrhythmia. The prevalence of AF is currently expected to significantly increase throughout the population of China by 2050, along with the elevated incidence of chronic diseases and changes in urban lifestyles and dietary habits [1]. AF leads to disturbed atrial rhythms, especially in the left atrium, creating a blood flow vortex [2]. Indeed, the left atrial appendage is the most common source of blood clots in 90% of non-valvular atrial fibrillation (NVAF) cases [3].

Recent research has shown a decrease in mortality from AF, with these improvements potentially related to preventing thromboembolic complications [4]. The annual incidence of embolic events in patients with NVAF is 5%, accounting for 15–20% of all cerebral embolisms [5]. Therefore, predicting cardiac thrombi is important. The left atrium is the most common source of thrombus in patients with NVAF. Transthoracic echocardiography (TTE) has poor specificity and sensitivity in diagnosing left atrial thrombus or spontaneous echo contrast (LAT/SEC) due to the influence of sound transmission conditions. Transesophageal echocardiography (TEE) is essential for diagnosing LAT/SEC [6].

Some scholars found that elevated body mass index (BMI) is related to a higher risk of AF and that the recurrent risk of AF is elevated in overweight and obese patients relative to those with a normal BMI [7]. This study aimed to analyze the impact of BMI on cardiac structure and function in cases with NVAF and examine the potential association between BMI and LAT/SEC incidence.

## 2. Methods

### 2.1 Study Design

**Fig. 1.**
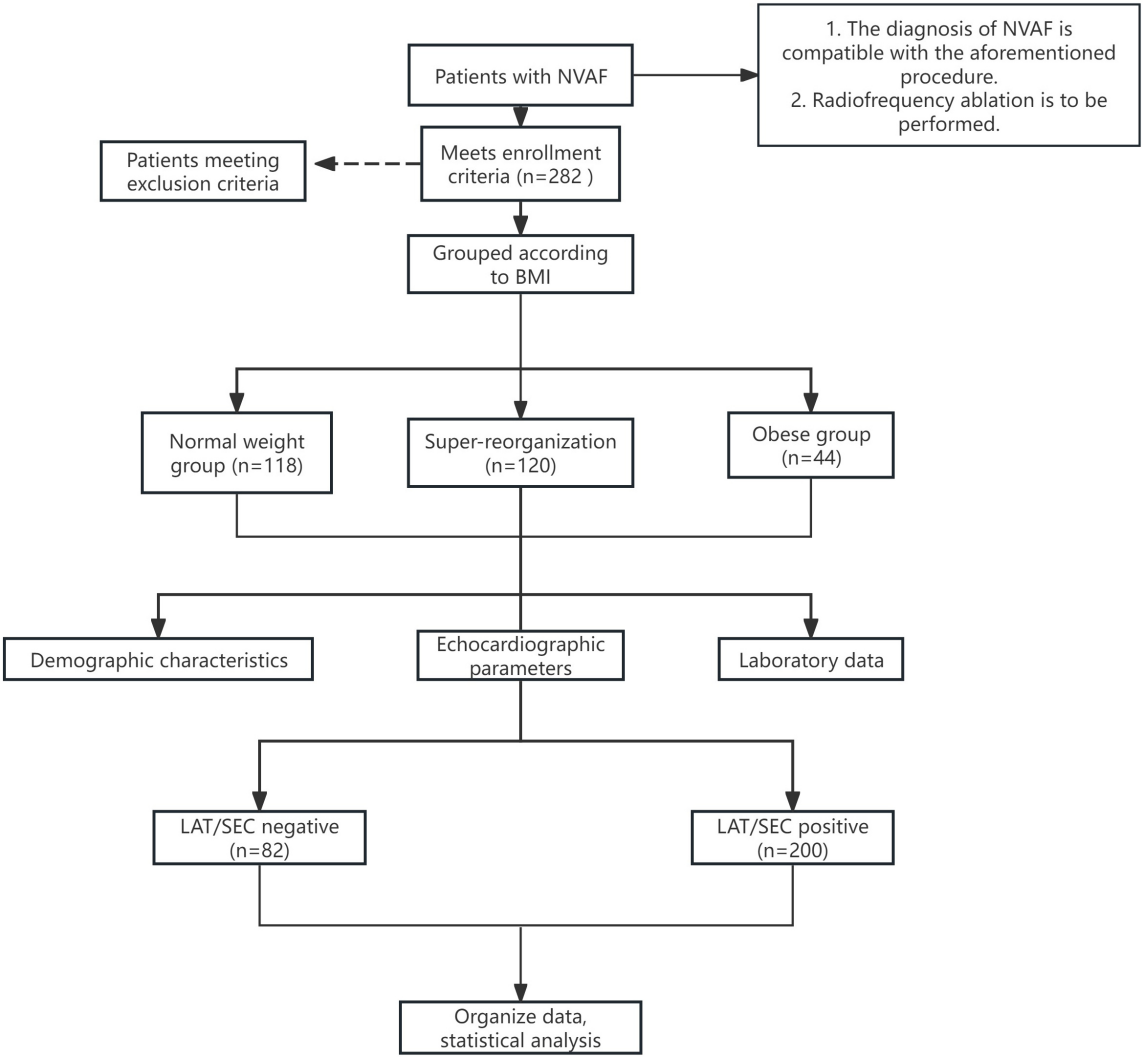
**Flowchart of the study population**. NVAF, non-valvular atrial fibrillation; BMI, body mass index; LAT, left atrial thrombus; SEC, spontaneous echo contrast.

This retrospective observational study recruited 282 patients aged 64.8 ± 9.4 years (mean ± SD). The highest and lowest ages were 84 and 29 years, respectively. The cases were recruited from our center from Jan 2019 to Dec 2022. All cases had a diagnosis of NVAF and were scheduled to undergo radiofrequency ablation. Inclusion criteria: (1) hospitalized patients definitively diagnosed with NVAF; (2) complete TTE and TEE during hospitalization. Exclusion criteria: (1) congenital heart disease, cardiomyopathy; (2) history of cardiac surgery; (3) use of a new oral anticoagulant or warfarin (International normalized ratio ≥2.0); (4) history of stroke. The flow of this study is shown in Fig. 1. This study was approved by the Ethics Committee of The First People’s Hospital of Changzhou and performed in line with relevant regulations.

### 2.2 Data Collection

(1) Data on comorbid disease, medications, and lifestyle factors were obtained. The participants underwent a standardized physical examination. The chronic conditions included diabetes and hypertension. Hypertension was defined as follows: systolic blood pressure (SBP) ≥140 mmHg and/or diastolic blood pressure (DBP) ≥90 mmHg. The following data were recorded for all patients: gender, age, height, weight, calculated BMI = (weight/height^2^), SBP, DBP, mean arterial pressure (MAP), and history of diabetes or hypertension. (2) Laboratory examination: The Laboratory Department at our center analyzed the biochemical indicators. All blood samples were centrifuged within an hour of collection, after which the serum was separated and analyzed. Systemic levels of uric acid (UA), total cholesterol (TC), triglycerides (TGs), low-density lipoprotein (LDL)-cholesterol (LDL-C), high-density lipoprotein (HDL)-cholesterol (HDL-C), apolipoprotein A1 (ApoA) and apolipoprotein B (ApoB), were determined was using colorimetric assays. (3) TTE was used to obtain aortic root diameter (AOD), left atrial diameter (LAD), left ventricular end-diastolic diameter (LVEDD), interventricular septal thickness (IVST), left ventricular posterior wall thicknes (LVPWT), left ventricular ejection fraction (LVEF), and right atrial diameter (RAD); (4) TEE was required to identify LAT/SEC on at least two or more cross-sectional images. The LAT was defined as an echogenic mass with a distinct texture and uniform consistency, differing from the left atrial wall. SEC was recorded when the left atrial appendage exhibited weak echoes, moderate echoes in a dense, swirling pattern, and high echoes in a slow, swirling pattern [8].

### 2.3 Statistical Analysis

Continuous variables were expressed as the mean ± SD. Categorical data were expressed as numbers and ratios. The Mann–Whitney and chi-square tests were applied to test for statistical differences in the two groups. A multivariate logistic regression model was used to analyze the relationship between BMI and LAT/SEC. The non-adjusted and simple adjusted model (adjusted for age, MAP) and multivariate-adjusted models (adjusted for age, MAP, UA, ApoA, LAD, LVEDD, IVST, LVEF, RAD) were used to analyze the relationship between BMI and LAT/SEC. The generalized additive model (GAM) was used to analyze the dose-response association between BMI and the occurrence of LAT/SEC. All analyses were conducted using statistical software R (http://www.R-project.org, R version 4.2.0) and EmpowerStats (http://www.empowerstats.com, 2011 X&Y Solutions, Inc., Boston, MA, USA). A *p*-value < 0.05 was regarded as statistically significant.

## 3. Results 

### 3.1 Demographic Characteristics

Data from 282 cases were analyzed. The median age was 66 years (IQR (interquartile range): 29–84 years). A total of 131 cases (46.5%) were females. BMI was used to categorize patients into a normal weight group (18.5 ≤ BMI <24 kg/m^2^), overweight group (BMI: 24–28 kg/m^2^), and obese group (BMI: ≥28 kg/m^2^). Table 1 shows the basic information of the BMI groups. Patient demographics, vital signs, laboratory findings, and echocardiographic parameters were compared according to BMI category (Table 1).

**Table 1.** **Baseline information according to BMI (n = 282)**.

Parameters	BMI			*p*-value
	Normal weight (118)	Overweight (120)	Obese (44)	
Demographic characteristics				
Age (year)	66.88 ± 8.28	63.68 ± 9.41	62.57 ± 11.23	0.014
Sex				
Male (%)	63 (53.3)	69 (57.5)	19 (43.2)	0.265
Female (%)	55 (46.7)	51 (42.5)	25 (56.8)	
Diabetes (%)	16 (13.6)	17 (14.2)	12 (27)	0.082
Hypertension (%)	66 (55.9)	79 (65.8)	32 (72.7)	0.095
MAP (mmHg)	92.66 ± 10.32	95 ± 9.46	98.39 ± 11.67	0.005
Laboratory data				
UA (µmol/L)	324.57 ± 104.43	370.46 ± 90.78	384.38 ± 94.01	0.000
TC (mmol/L)	4.49 ± 1.47	5.24 ± 0.23	4.65 ± 0.95	0.146
TG (mmol/L)	1.43 ± 0.65	1.77 ± 1.03	1.84 ± 0.85	0.003
HDL-C (mmol/L)	1.20 ± 0.27	1.05 ± 0.23	1.00 ± 0.24	0.000
LDL-C (mmol/L)	2.51 ± 0.79	2.46 ± 0.69	2.81 ± 0.80	0.047
ApoA (g/L)	1.28 ± 0.23	1.19 ± 0.20	1.17 ± 0.22	0.004
ApoB (g/L)	0.87 ± 0.25	0.87 ± 0.24	0.98 ± 0.26	0.035
Echocardiographic parameters				
AOD (mm)	31.86 ± 4.06	32.22 ± 3.39	32.18 ± 3.64	0.420
LAD (mm)	39.58 ± 5.95	43.66 ± 5.97	43.16 ± 4.59	0.000
LVEDD (mm)	47.16 ± 4.13	50.06 ± 4.31	51.11 ± 4.49	0.000
IVST (mm)	9.23 ± 1.60	9.61 ± 1.06	9.57 ± 0.97	0.000
LVPWT (mm)	8.96 ± 0.71	9.49 ± 0.92	9.48 ± 0.85	0.000
LVEF (%)	62.01 ± 5.02	59.58 ± 5.78	61.64 ± 4.48	0.001
RAD (mm)	37.93 ± 5.99	40.63 ± 7.61	38.97 ± 5.71	0.008

Data were described as the mean ± SD, median, or percentage. For the included cases, the number of missing values was 0. BMI, body mass index; MAP, mean arterial pressure; UA, uric acid; TC, total cholesterol; TG, triglyceride; HDL-C, high-density lipoprotein-cholesterol; LDL-C, low-density lipoprotein-cholesterol; ApoA, apolipoprotein A1; ApoB, apolipoprotein B; AOD, aortic root diameter; LAD, left atrial diameter; LVEDD, left ventricular end-diastolic diameter; IVST, interventricular septal thickness; LVPWT, left ventricular posterior wall thickness; LVEF, left ventricular ejection fraction; RAD, right atrial diameter.

### 3.2 Incidence Rate of LAT/SEC

**Fig. 2.**
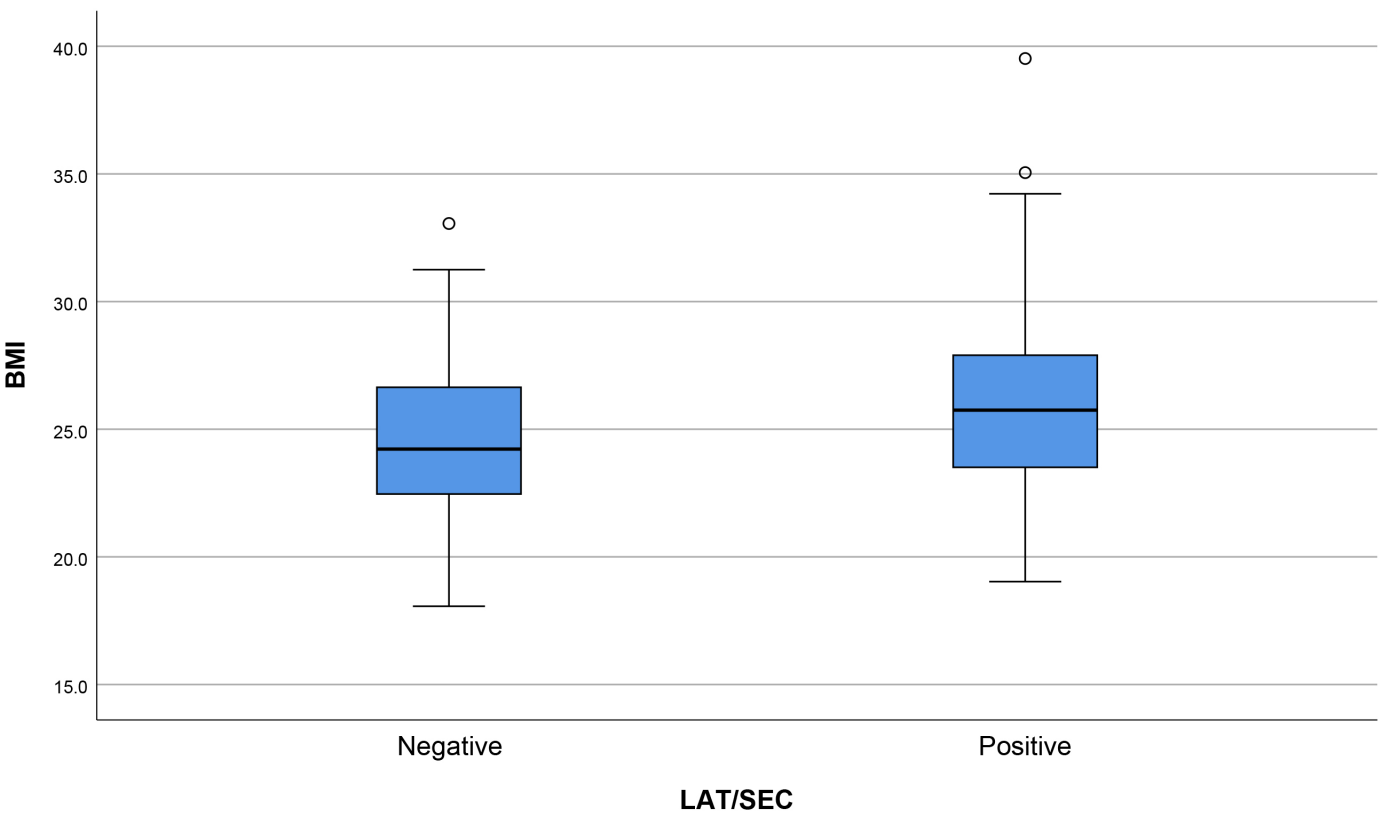
**Comparison of body mass index (BMI) in cases with or without left atrial thrombus (LAT)/spontaneous echo contrast (SEC)**.

A total of 24 cases (20.3%) relating to LAT/SEC incidence were observed in the normal weight group, with 38 cases (31.7%) in the overweight group and 20 cases (45.5%) in the obesity group. Box plots of BMI distribution after grouping according to the presence or absence of LAT/SEC are shown in Fig. 2.

### 3.3 Relationship between BMI and LAT/SEC

**Fig. 3.**
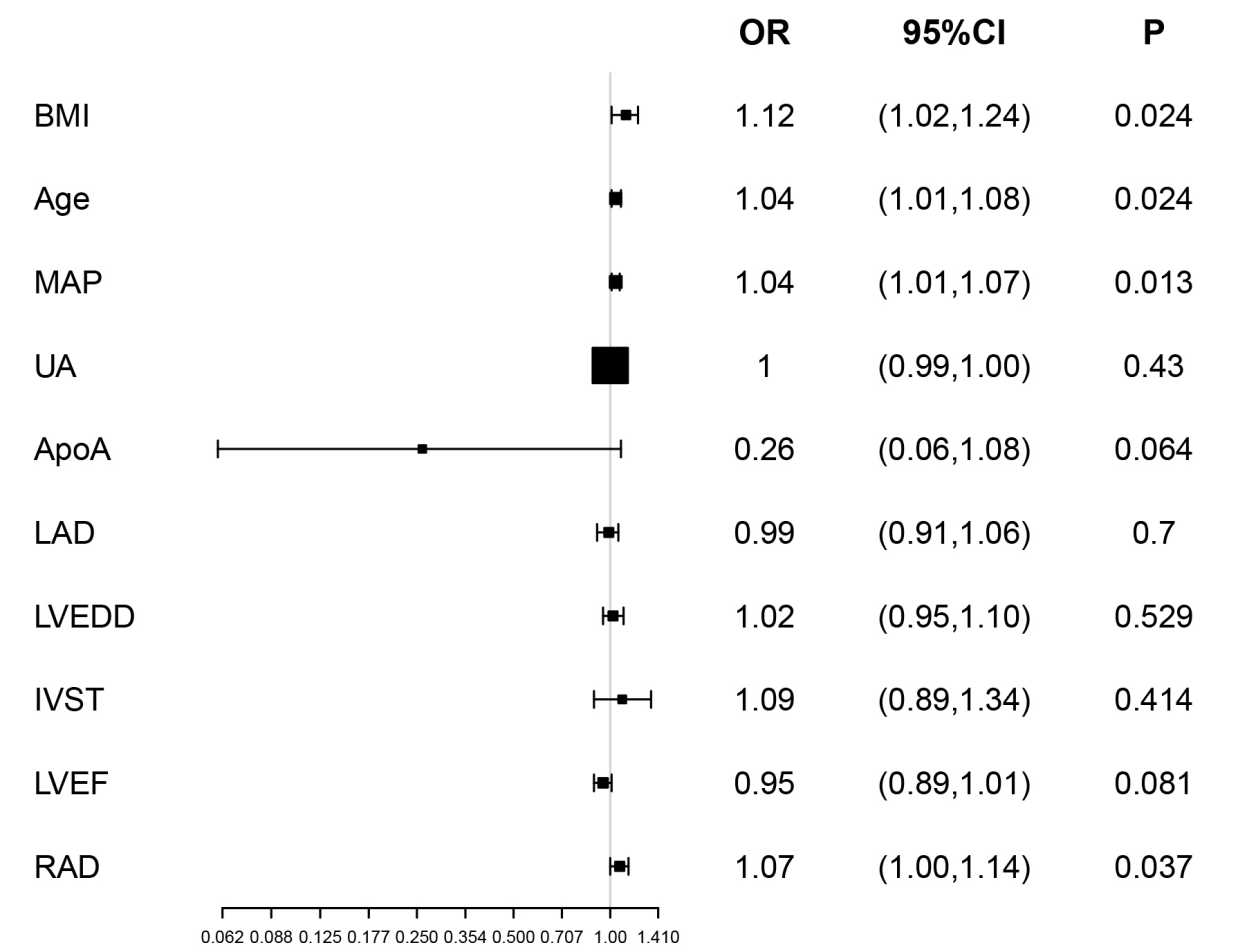
**Risk ratios for LAT/SEC, according to baseline characteristics**. LAT, left atrial thrombus; SEC, spontaneous echo contrast; BMI, body mass index; MAP, mean arterial pressure; UA, uric acid; ApoA, apolipoprotein A1; LAD, left atrial diameter; LVEDD, left ventricular end-diastolic diameter; IVST, interventricular septal thickness; LVEF, left ventricular ejection fraction; RAD, right atrial diameter; CI, confidence interval; OR, odds ratio.

The association between BMI and LAT/SEC is shown in Table 2. Univariate logistic analysis (Model 1) showed that BMI was related to LAT/SEC (odds ratio (OR) = 1.16; *p* < 0.001). Variables were adjusted for age and MAP (Model 2), which did not notably change the results (OR = 1.18; *p* < 0.001). Likewise, there was no significant change in results after fully adjusting for all covariates (age, MAP, UA, ApoA, LAD, LVEDD, IVST, LVEF, RAD; Model 3) (OR = 1.12; *p *= 0.024). The results of these multiple factor logistic analyses are shown in Fig. 3.

**Table 2.** **An evaluation of the association between BMI and LAT/SEC using multinomial logistic regression models**.

	OR	95% CI	*p*-value
Model 1 (unadjusted)			
LAT/SEC negative	Reference		
LAT/SEC positive	1.16	1.07, 1.26	0.0002
Model 2 (adjusted for age, MAP)			
LAT/SEC negative	Reference		
LAT/SEC positive	1.18	1.08, 1.29	0.0003
Model 3 (adjusted for age, MAP, UA, ApoA, LAD, LVEDD, IVST, LVEF, RAD)			
LAT/SEC negative	Reference		
LAT/SEC positive	1.12	1.02, 1.24	0.024

Model 1 was unadjusted. Model 2 was adjusted for age and MAP. Model 3 was adjusted for age, MAP, UA, ApoA, LAD, LVEDD, IVST, LVEF, and RAD. CI, confidence interval; OR, odds ratio; BMI, body mass index; MAP, mean arterial pressure; UA, uric acid; ApoA, apolipoprotein A1; LAD, left atrial diameter; LVEDD, left ventricular end-diastolic diameter; IVST, interventricular septal thickness; LVEF, left ventricular ejection fraction; RAD, right atrial diameter; LAT, left atrial thrombus; SEC, spontaneous echo contrast.

### 3.4 Dose-Response Association between BMI and LAT/SEC

**Fig. 4.**
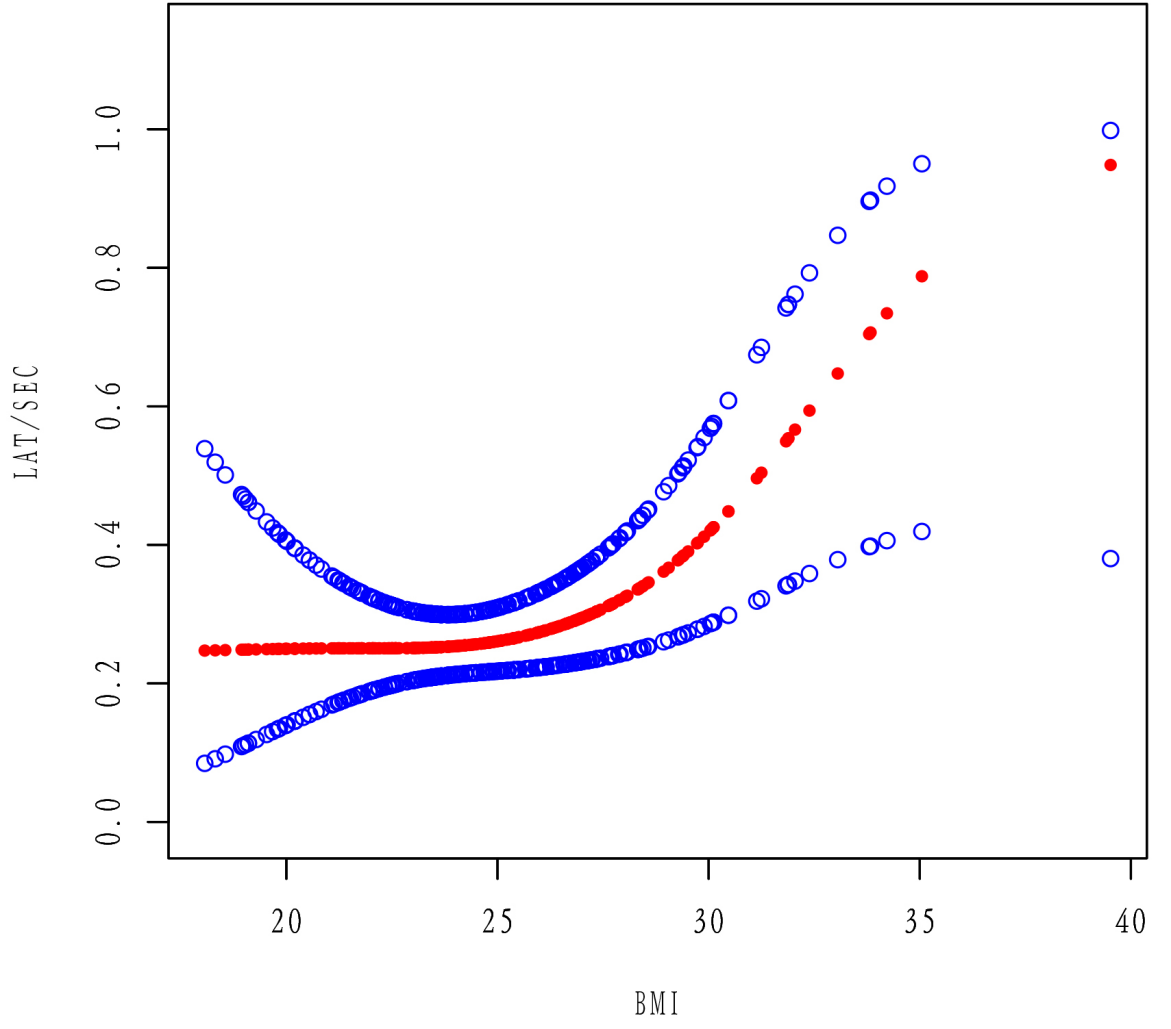
**Associations between BMI and LAT/SEC incidence in all patients with NVAF**. The red dotted line corresponds to the smooth curve between the variables. Values were adjusted for age, MAP, UA, ApoA, LAD, LVEDD, IVST, LVEF, and RAD. LAT, left atrial thrombus; SEC, spontaneous echo contrast; BMI, body mass index; MAP, mean arterial pressure; UA, uric acid; ApoA, apolipoprotein A1; LAD, left atrial diameter; LVEDD, left ventricular end-diastolic diameter; IVST, interventricular septal thickness; LVEF, left ventricular ejection fraction; RAD, right atrial diameter; NVAF, nonvalvular atrial fibrillation.

A nonlinear dose-response association exists between BMI and LAT/SEC (Fig. 4). The risk of an incidence of LAT/SEC was 1.98 times higher with a BMI of ≥27 kg/m^2^ than that with a BMI of <27 kg/m^2^.

## 4. Discussion

As a consequence of the increased aging of the global population, NVAF has become the most prevalent type of arrhythmia worldwide and a significant contributor to systemic embolism, resulting in increased direct and indirect costs to families and society overall [9]. LAT represents a significant etiological factor for cardiac embolism in patients with NVAF. Furthermore, the occurrence of a cardiac embolism serves as an independent risk factor for an ischemic stroke [10]. The presence of SEC is a harbinger of left atrial appendage thrombosis, which is linked to an increased risk of thromboembolism and major adverse cerebrovascular events. SEC can be utilized as a surrogate marker for thromboembolism from the left atrial appendage [11]. Therefore, our study selected a population-based endpoint (LAT/SEC) and investigated its correlation with BMI.

This research revealed that the differences between the three groups were primarily concentrated between the normal group and the overweight and obese groups. As BMI increased, there was a notable trend in increased UA and triglyceride levels, accompanied by a decline in HDL-C and ApoA1. Individuals with a high BMI demonstrated an imbalance in caloric intake and energy expenditure, resulting in excessive abdominal fat accumulation, enhanced overall nucleic acid metabolism, and the promotion of UA synthesis through purine metabolism. Obese individuals have an imbalance between calorie intake and energy expenditure, leading to the excessive accumulation of abdominal fat, and promoting uric acid synthesis by purine metabolism. Increased adipose tissue contributes to fat cytokine imbalance resulting in high insulin hematic disease, high blood insulin increased renal tubular reabsorption of sodium and uric acid, resulting in reduced uric acid excretion, which leads to hyperuricemia. Studies have also demonstrated that lowering fat mass causes lower serum UA levels [12, 13, 14]. Song *et al*. [15] found that increased UA, enlarged LA, and low LVEF are independent risk factors for increased stroke risk in patients with NVAF. Xanthine oxidase is responsible for converting purines into UA, which serves as the final product of purine metabolism. Oxygen free radicals generated during metabolic processes elevate oxidative stress levels in atrial tissues, thereby impeding nitric oxide production by endothelial cells and fostering tissue inflammation while exacerbating endothelial cell impairment. In addition, high levels of UA activate platelets *in vivo*, which promotes platelet adhesion and aggregation, leading to thrombus formation [16].

The present study revealed that LAD, LVEDD, and RAD tended to increase when the BMI reached the overweight and obese categories compared to the normal category. Conversely, the LVEF values tended to decrease in the overweight category compared to the normal category. In a prospective, observational, and multicenter study conducted by Uziębło-Życzkowska *et al*. [17], an elevated BMI was related to an enlarged LAD in NVAF cases based on data from 13 cardiology centers. A study of 203 NVAF subjects revealed a statistically significant increase in left atrial volume in the obese group compared to the other two groups [18]. A large-scale lifeline cohort study of health behaviors of 167,729 participants in the Netherlands determined that relative adiposity was related to the risk of AF (OR: 1.58, 95% CI: 1.33, 1.87) [19]. The mechanism through which left atrial enlargement occurs in overweight and obese NVAF patients is thought to be linked to the infiltration of epicardial fat in the myocardium. This infiltration results in autocrine activity that contributes to atrial myocardial structural remodeling. Additionally, the paracrine effect of secreted adipocytokines, which induce atrial fibrosis, is a contributing factor. Furthermore, obese individuals are in a state of high cardiac output, which promotes left atrial remodeling and diastolic dysfunction [20, 21]. An increase in body weight has been demonstrated to result in a deterioration of insulin resistance, accompanied by an elevation in inflammatory and oxidative stress. This, in turn, leads to endothelial dysfunction and cardiomyocyte apoptosis, which impairs myocardial contractility and ultimately leads to a decline in myocardial function [22]. In the overweight and obese groups, a higher BMI tended to promote a decrease in the LAD and RAD. This phenomenon is attributed to the accumulation of pericardial fat, which creates a mechanical obstacle that compresses the left atrium, thereby restricting the dilatation of the heart while impairing the mobility of the left ventricular sidewalls, leading to diastolic dysfunction [23].

The findings of this study indicate that LAT/SEC was observed in 82 out of 282 NVAF cases (29.1%). Previous research has proposed that LAT/SEC may occur in 10–60% of patients with NVAF [24]; thus, our data aligns with prior research. The correlation between BMI and LAT/SEC was tested using a GAM. The results indicated that the correlation was not significant at a BMI <27 kg/m^2^; however, it was significantly correlated when the BMI was ≥27 kg/m^2^. Conducting the grouping using a BMI of 27 kg/m^2^ as the boundary revealed that the incidence of LAT/SEC with a BMI of ≥27 kg/m^2^ was 1.98 times higher than that with a BMI of <27 kg/m^2^. This suggests the effect on cardiac LAT/SEC becomes significant after the BMI exceeds this threshold. Obesity is regarded as a chronic low-grade inflammatory state. The presence of high pro-inflammatory markers such as leptin and interleukin-6 (IL-6), and low anti-inflammatory markers such as lipocalin, indicates high levels of proinflammatory markers, such as leptin and IL-6, and low anti-inflammatory markers, such as lipocalin, indicate that these markers may be associated with oxidative stress and structural remodeling of the atria [25]. The presence of hyperlipidemia in cases with obesity is related to a higher risk of platelet hyperactivity. Inflammatory stimuli exert a significant function in the progression of endothelial damage and hypercoagulation, increasing the likelihood of a prethrombotic state [20]. Jian Li *et al*. [26] enrolled 102 NVAF subjects and divided them into two groups based on thrombus. They observed an obvious difference in the LVEF between the two groups (0.57 ± 0.1 *vs*. 0.61 ± 0.08, *p* = 0.035); similar results were found in our study. A reduction in the LVEF expands the left end-diastolic volume, increasing left atrial pressure and volume. As the volume of the left atrium increases, left atrial appendage compliance rises, and the left atrial appendage pressure load and volume load increase, leading to alterations in blood flow velocities and the formation of thrombi [26]. Indeed, recent evidence suggests that, as noninvasively assessed by speckle tracking echocardiography, left atrial dysfunction may indirectly reveal left atrial appendage dysfunction, thus predicting LAT/SEC in NVAF patients. The lower the left atrial reservoir strain magnitude, the higher the probability of LAT/SEC [27].

It has been demonstrated that either being overweight or obese is related to a heightened likelihood of higher risks of new-onset AF (12.3% *vs*. 32.7%) and a higher rate of recurrence of AF. This is attributed to various neurohumoral and metabolic alterations, such as increased insulin resistance, renin–angiotensin–aldosterone system stimulation, and elevated arterial hypertension [28].

The increased BMI in the NVAF results in significant cardiac structure and function changes. Obesity is a manageable adverse factor; therefore, it is vital to emphasize the importance of nutritional education and dietary interventions to limit the occurrence and reduce the complications of AF.

However, this study was constrained by its limited sample size and potential bias. Furthermore, the classification of obesity requires refinement to facilitate individualized management and guidance for different types of obesity and to limit adverse thrombotic outcomes in the NVAF population.

## 5. Conclusions

BMI significantly affects multiple echocardiographic parameters in patients with NVAF, and BMI is an independent risk factor for LAT/SEC in NVAF.

## Availability of Data and Materials

The data that support the findings of this study are available on request from the corresponding author.
